# Strategic Bidding of Retailers in Wholesale Markets: Continuous Intraday Markets and Hybrid Forecast Methods †

**DOI:** 10.3390/s23031681

**Published:** 2023-02-03

**Authors:** Hugo Algarvio, Fernando Lopes

**Affiliations:** LNEG–National Laboratory of Energy and Geology, Est. Paço Lumiar 22, 1649-038 Lisbon, Portugal

**Keywords:** wholesale markets, continuous intraday markets, retailers, forecast methods, consumers

## Abstract

The deregulation process of the electricity sector has led to competition in wholesale and retail markets. In particular, retailers submit bids to wholesale markets to satisfy the energy needs associated with portfolios of end-use customers. This paper describes a strategic process for retailers bidding in a wholesale market composed of a day-ahead market, an intraday market, and a balancing market. It considers a market design that involves a hybrid model for the intraday market, based on daily auctions and a continuous procedure. The paper also presents a computational study to illustrate and test both the market design and the strategic bidding process of retailers. The results confirm the advantages of considering a continuous intraday market, show that bidding in short-term markets is more beneficial than bidding in medium-term markets, and indicate important aspects to consider when selecting customers to add to the portfolios of retailers.

## 1. Introduction

The liberalization process has led to competition in wholesale and retail markets [1]. Market players now have the option to trade electricity in three different (sub-)markets: spot, bilateral, and non-organized markets (private bilateral contracts). In spot markets, players can submit bids to electricity pools based on day-ahead and intraday auctions. In bilateral markets, players can sign standard financial and physical contracts to hedge against spot price volatility. In non-standard markets, players can negotiate privately and set the terms and conditions of bilateral contracts (see, e.g., [2,3]).

Wholesale markets were designed when the vast majority of generation units were controllable. Thus, market players that experience difficulties in complying with a dispatch procedure may incur real-time deviations from schedules. Such players—called balance responsible parties (BRPs)—may need to pay/receive the down/up balancing costs [4]. For this reason, and also other important reasons, there is a growing need to adapt market rules to the new market realities (see [5,6,7]). In 2019, the European Commission published new rules for the internal market for electricity [7,8,9]. For wholesale markets, the new rules focus on several aspects, including short term markets to improve competition and liquidity. The lead time—that is, the time between the end of a trading session and the start of a delivery period—is a key issue [10]. Ideally, it should be lower than or equal to 1 h (e.g., 5, 15, 30 or 60 min). Another key issue is the structure and organization of the intraday market. Traditionally, this market operates by considering several daily sessions based on auctions. However, in the past few years, the EU published new operational rules for the intraday market [11]. Among other aspects, the rules define a continuous trading procedure throughout the day, which can be complemented with regional intraday auctions. This raises important questions (e.g., ‘Should EU countries consider a continuous intraday market only?’, ‘Should they complement the continuous market with intraday auctions?’, and ‘How to integrate the continuous market with the auctions?’).

In retail markets, retailers can sign bilateral contracts with consumers [12]. They usually follow a business-as-usual strategy, proposing high tariffs that are equal in each consumer segment. Additionally, they often consider a high-risk premium, meaning that the energy element of tariffs is substantially higher than the wholesale price of electricity. Such a risk premium depends on the risk attitude (also known as risk preference or risk appetite), which can be characterized as risk-averse, risk-neutral and risk-seeking. To avoid unexpected losses, a key aspect that retailers need to consider when proposing tariffs to consumers is the forecast of market prices [13]. Another important aspect is the forecast of the energy needs associated with their portfolios. Consumption variability is dependent on meteorological conditions, consumption days (weekdays, holidays or weekends), and type of consumers (residential, commercial or industrial). Minimizing this variability is a good measure to avoid errors associated with bids submitted to the wholesale market, which can result in the payment of penalties. Thus, retailers should consider an appropriate process to mitigate such errors [14].

This paper focuses on both market design and the strategic bidding process of retailers. It extends the work presented in [15] by considering the following: (i) a continuous intraday market, where players can negotiate 1h-ahead of real-time, and (ii) a slightly updated forecast method for submitting bids to the intraday market. It considers a wholesale market composed of a day-ahead (sub-)market, an intraday (sub-)market, and a balancing (sub-)market. The intraday market is assumed to operate continuously, seven days a week, all year around. This market is also assumed to involve six daily sessions based on auctions. The rationale for this assumption is simply to consider the traditional operation of the Iberian intraday market. Thus, for simplicity, we consider a market design based on the traditional Iberian market design, extended with a continuous intraday market. We note, however, that future work will investigate new market designs based on different intraday auctions and their close interaction with the continuous intraday market. We also note that future work will examine market design from different points of view, i.e., other than that of retailers (as this work does). This includes, naturally, the perspective of renewable energy producers. The purpose of the paper is threefold:

1. To analyse a hybrid model for the intraday market, based on daily auctions and a continuous procedure.
2. To analyse a strategic process for retailers submitting bids to the wholesale market.
3. To present a computational study that illustrates and tests both the aforementioned market design and the strategic bidding process of retailers; the study involves six retailer agents with different risk attitudes.

The work presented here refines and extends our previous work on electricity markets [7,10], portfolio optimization [12,13], risk management [3,14,16], and strategic bidding [15]. The experimental work was carried out with the help of the MATREM system [17]. The remainder of the paper is structured as follows. Section 2 presents a literature review of the strategic bidding of retailers in wholesale markets. Section 3 presents an overview of bilateral contracting, risk management and portfolio optimization. Section 4 describes a model for the strategic bidding of retailers. Section 5 presents the computational study. Finally, concluding remarks are presented in Section 6.

## 2. Literature Review

Hatami et al. [18] developed a decision support framework for retailers that need to buy electricity to satisfy their customers. The main goal of retailers was to determine the best tariffs by considering different options, notably spot markets, forward contracts, call options and self-generating facilities. The optimization process aimed at increasing profit and reducing the risk inherent to portfolios. The authors considered a fixed number of clients, using a fixed demand and the associated elasticity. They did not consider real consumption data. Hatami et al. [19] extended this work by considering interrupted contracts and an elasticity matrix that considers different load elasticities for each time-of-use block.

Kettenun et al. [20] described retailers with different risk attitudes to obtain an optimal portfolio of forward contracts. The retailers faced risks associated with spot price volatility and consumption variability. The authors considered a strong correlation between spot prices and the demand of retailers, which can be true for large retailers, but not necessarily for all retailers. Their study considered the demand from the whole Nord Pool, a pan-European power exchange, meaning that the effectiveness of the model was not tested with typical retailers. They concluded that risk-neutral retailers are more concerned with price-related uncertainty, while risk-averse retailers with forward contracts hedge against spot price volatility. Nojavan et al. [21] presented an optimization model to minimize the electricity cost of retailers in wholesale markets. The authors considered a day-ahead market, forward contracts, and demand–response programs. They concluded that risk-averse retailers tend to trade large quantities of energy by signing forward contracts as a risk mitigation measure to hedge against the volatility of the day-ahead price.

Ayón et al. [22] indicated that large and non-uniform portfolios of end-use consumers may reduce demand forecast errors. The authors also stated that aggregations of flexible demand are typically beneficial to reduce demand forecast errors, increasing the return of aggregators. Wei et al. [23] presented a complete review of 128 forecast models for energy demand. They considered a mean absolute percentage error (MAPE) of 10% as a threshold for highly accurate forecasts. They concluded that small-scale forecasts (e.g., a consumer or building) have larger errors than large-scale forecasts (e.g., national level consumption). They also concluded that the forecast accuracy increases with the time horizon—that is, long-term (yearly) and medium-term (monthly or quarterly) forecasts have smaller errors than short-term forecasts (daily or sub-hourly). Koponen et al. [24] presented a review of 12 models to forecast the short-term electricity demand. The authors used these models in six different scenarios. They concluded that the normalized root mean square error (NRMSE) of the forecast decreases with the number of aggregated consumers. They also stated that hybrid methods should be used to compute demand forecasts.

Shah et al. [25] compared different demand and price forecast approaches. The authors considered the following two components in their forecasting methodology: a deterministic component based on parametric and non-parametric approaches, and a stochastic component based on univariate and multivariate vector autoregressive models. The autoregressive models were estimated using the following four methods: least squares, Lasso, Ridge, and Elastic-net. They tested these approaches in the day-ahead market of the North-European power exchange. They concluded that a multivariate vector autoregressive model using an Elastic-net estimator leads to the lowest demand forecast errors with a MAPE of 2.017%.

## 3. Bilateral Contracting, Risk Management and Portfolio Optimization

Sellers and buyers of electricity negotiate bilateral contracts to hedge against spot prices volatility. Standard bilateral contracts can be physical or financial and include the following [3,5]: forwards, futures, options, and swaps (also known as contracts for differences). For non-standard contracts, several terms and conditions can be negotiated, such as energy price, energy quantity and duration (see, e.g., [2]). Although these contracts are used for risk hedging, they also involve some risk.

Sellers and buyers can follow a risk management process in order to select the best options to trade electricity, which involves the following three phases [26,27]:

- Risk assessment phase: players recognize the risk factors and identify the deterministic and stochastic variables.
- Risk characterization phase: risk is measured using different methods, such as correlation, regression, value-at-risk (VaR), or conditional VaR (CVaR); VaR measures the potential losses of investors to a certain degree of confidence in a given time interval; CVaR has a higher dimension than VaR, because it considers the case when the worst scenario is surpassed; VaR only computes the expected loss of the worst scenario.
- Risk mitigation phase: players select the best set of market products to reduce the risk of their transactions.

Retailers aim to optimize the risk–return output of their portfolios. To this end, they sign bilateral contracts with key consumers and select the best set of market options to trade energy. In [12], we introduce an optimization model to select key consumers for the portfolios of retailers. The model constitutes a basis for the present (conceptual) work and we describe some important aspects of it in the following section. The model considers the three aforementioned phases of the risk management process and the Markowitz theory to define an efficient frontier. In the first phase, retailers face the following risk factors: market price volatility and consumption variability. In the second phase, retailers use the VaR to analyse how these risk factors can affect their return. In the third phase, retailers optimize the share of consumers in their portfolios by considering their attitude towards risk and the electricity tariff proposed to consumers. The efficient frontier is defined by considering the risk factors defined in the first phase, as well as the risk analysis carried out in the second phase and the efficient points obtained in the third phase. Retailers obtain the efficient frontier from the efficient points (a particular point is considered efficient if no other point can surpass its result in terms of risk or return). The electricity tariff involves two parts, namely a fixed price for contracted power, and a variable price per unit of consumed electricity (which, in turn, is divided into several parts). Notice that the most common tariffs for household consumers are single tariffs (equal during all day-periods), two-rate tariffs (involving peak and off-peak), three-rate tariffs (involving peak, intermediate and off-peak), and four-rate tariffs (involving peak, intermediate, off-peak and super off-peak). Commercial and industrial consumers connected to high or very-high voltage grids can also choose other tariffs.

Additionally, in [12], we present several strategies that can be adopted by retailers to negotiate with customers. In particular, the “Equal Return OPtimization” strategy (EROP) defines the minimum tariff that retailers may offer to each consumer to receive an equal target return from each one. The “Equal Tariff Optimization at a Minimum Return” strategy (ETOMinR) is not personalized, meaning that it considers the same tariff for all consumers. Retailers compute a tariff for each consumer and select the highest one because it guarantees a minimum target return. The “Equal Tariff Optimization at a Maximum Return” strategy (ETOMaxR) is also not personalized. To guarantee a maximum target return from all consumers, retailers compute a tariff for each consumer, and the minimum tariff between all the computed tariffs is selected. This pricing strategy may be used when regulators define a maximum return that retailers cannot surpass. The “Equal Return Tariff based on Market-Costs” strategy (ERTMC) reflects the expected costs of retailers with each consumer, considering their consumption patterns. These strategies can be selected by considering the type of tariff (i.e., two-rate, three-rate, four-rate, and so on), the equality of tariff (i.e., personalized or not), and the equality of return (i.e., if each consumer presents a similar return for retailers).

The forecast method presented in [12], and adopted in this work, consists of a Multivariate Time series (MTS) that uses the wholesale market prices of electricity, the electricity consumption, and the share of renewable energy associated with electricity production to predict the arithmetic cost of electricity for each consumer. Additionally, future predictions of electricity consumption are computed by adopting an MTS forecast method from [13], involving electricity consumption, retail electricity prices, and gross domestic products. The so-called Calinski–Harabasz (CH) criterion computes the Euclidean distance between different clusters and compares it with the internal sum of squared errors for each cluster. The optimal number of profiles (clusters) is obtained by considering the consumption profile of each consumer and using the CH criterion. Each cluster represents the consumption segment of each consumer, with a typical load profile. The division of consumers by profile is computed by using K-means clustering, a robust algorithm that minimizes the distance between each point to the centre of its respective cluster.

## 4. Strategic Bidding in Wholesale Markets

**Figure 1:**
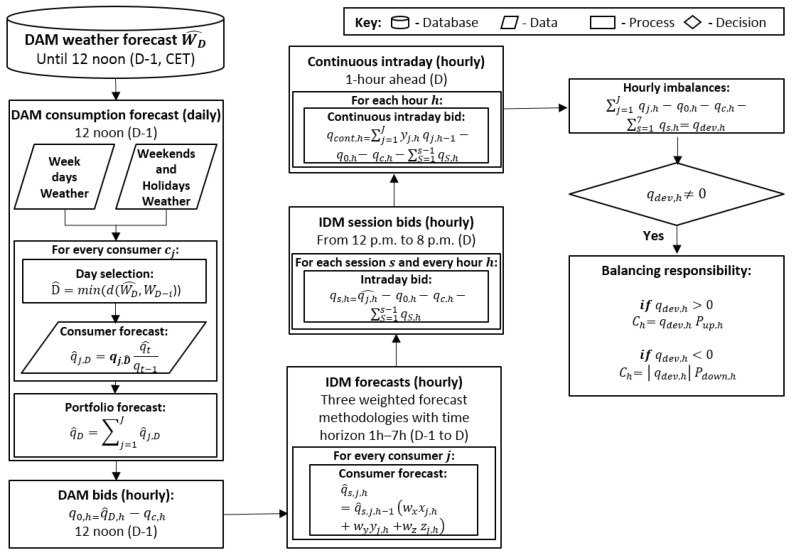
Strategic bidding process of retailers in wholesale markets.

Retailers submit bids to the spot day-ahead (DAM) and intraday markets (IDM), and to the continuous intraday market. The day-ahead market is used to buy/sell potential needs/excesses of energy. Each session of the IDM can be used to compensate for the expected imbalances between the energy traded and consumed. The continuous intraday market can be used to make adjustments close to real-time operation. Additionally, retailers can sign bilateral contracts to acquire energy to satisfy the needs associated with their portfolios. This section outlines the strategic bidding process of retailers (see also Figure 1). For convenience, the process is described as a sequence of steps (step 1 to step 7).

1. Retailers determine the consumption forecast for guiding the submission of bids to the DAM. For each consumer, *j*, they select the past day, $\hat{D}$, with the minimum Euclidean distance, *d*, between the historical weather data of a particular past day, $W_{D-i}$, and the weather forecast of the target day, ${\hat{W}}_{D}$, by considering one or more weather variables (e.g., temperature and humidity):
  (1) $$\hat{D}\;=\;\;min\left(d\left({\hat{W}}_{D},W_{D-i}\right)\right)$$
  They compute the expected consumption, ${\hat{q}}_{j,D}$, by determining the consumption of the past day, $q_{j,\hat{D}}$, and comparing the consumption forecast of the current year, ${\hat{q}}_{t}$, with the consumption of the past year $q_{t-1}$:
  (2) $${\hat{q}}_{j,D}\;=\;\;q_{j,\hat{D}}\frac{{\hat{q}}_{t}}{q_{t-1}}$$
  They compute the hourly consumption forecast of the portfolio, ${\hat{q}}_{D,h}$, by adding the forecasts of each consumer for each time period, *h*, of day *D*:
  (3) $${\hat{q}}_{D,h}\;=\;\;\sum_{h=1}^{H}\sum_{j=1}^{J}{\hat{q}}_{j,D,h}$$
  where ${\hat{q}}_{j,D,h}$ is the forecast of consumer *j*.
2. Retailers determine the bids submitted to the DAM by taking into account the energy of the whole portfolio, $q_{0,h}$. For each time period, they can sign several contracts, Ct, involving an energy quantity, $q_{c,h}$, so that:
  (4) $$q_{0,h}\;={\hat{q}}_{D,h}-q_{c,h}$$
  (5) $$q_{c,h}\;=\sum_{ct=1}^{Ct}q_{c_{ct},h}$$
  where $q_{c_{ct},h}$ is the energy associated with contract ct. For a particular time period, in case the quantity of energy guaranteed through bilateral contracts, $q_{c,h}$, is higher than the energy forecasted, ${\hat{q}}_{D,h}$, retailers try to sell the excess in a specific session of the the IDM. The associated bids are defined by following a simple strategy. If retailers are buying energy they offer the price-cap (maximum price) of the market to guarantee that they buy the energy required to satisfy the needs associated with their portfolios. Otherwise, they offer the price of the bilateral contracts to avoid economic losses.
3. Retailers compute the consumption forecast, ${\hat{q}}_{s,j,h}$, for bidding at each session, *s*, of the IDM, by considering a procedure that considers the meteorological conditions, the bids submitted to the DAM, $x_{j,h}$, the growth-rate method for a short-run period of 1 to 7 h, $y_{j,h}$, and the average consumption behaviour of each consumer between adjacent time periods, $z_{j,h}$:
  (6) $${\hat{q}}_{s,j,h}\;=\;\;{\hat{q}}_{s,j,h-1}\times \left(\sum_{h=1}^{H}\sum_{j=1}^{J}w_{x}x_{j,h}+w_{y}y_{j,h}+w_{z}z_{j,h}\right)$$
  where *j* denotes a specific consumer and *J* the total number of consumers. For time period, *h*, this formula considers the real consumption of the previous time period, if it exists, or its forecast, ${\hat{q}}_{s,j,h-1}$, and also the weight, *w*, of a particular method from a set of three methods. One method considers the bids submitted to the DAM, ${\hat{q}}_{j,D,h-1}$ and ${\hat{q}}_{j,D,h}$, at time periods h−1 and *h*, respectively:
  (7) $$x_{j,h}\;=\;\;\frac{{\hat{q}}_{j,D,h}}{{\hat{q}}_{j,D,h-1}}$$
  Another method considers the traditional behaviour of each consumer (obtained by analyzing the historical consumption data):
  (8) $$y_{j,h}\;=\;\;\frac{q_{\bar{j},h}}{q_{j,\bar{h}-1}}$$
  The last method considers the traditional behaviour and the last consumption tendency (to increase/decrease) of each consumer:
  (9) $$z_{j,h}\;=\;\;0.5*\left(y_{j,h}+\frac{q_{j,h-t}}{q_{j,h-t-1}}\right)$$
4. Retailers compute the bids to submit to each intraday session, $q_{s,h}$, at time period, *h*, by considering the short-run forecasts and all energy traded in the DAM, $q_{0,h}$, in the previous intraday sessions, $q_{i,h}$, and by bilateral contracts, $q_{c,h}$:
  (10) $$q_{s,h}\;=\;\;{\hat{q}}_{j,h}-q_{c,h}-q_{0,h}-\sum_{i=1}^{s-1}q_{i,h}$$
  where *s* denotes a specific intraday session.
5. Retailers compute the bids to submit to the continuous intraday market. The energy price, $P_{cont,h}$, is assumed to be the day-ahead price, $P_{0,h}$. The energy quantity, $q_{cont,h}$, is computed as follows:
  (11) $$q_{cont,j,h}\;=\;\;q_{j,h-1}\times y_{j,h}-q_{c,h}-q_{0,h}-\sum_{s=1}^{S}q_{s,h}$$
6. Retailers compute the imbalances, $q_{dev,h}$, for time period *h* by considering the real-time consumption of each customer of the portfolio, $q_{j,h}$:
  (12) $$q_{dev,h}\;=\;\;\sum_{j=1}^{J}q_{j,h}-q_{cont,j,h}-\sum_{s=1}^{S}q_{s,h}-q_{c,h}-q_{0,h}$$
7. Retailers compute their balance responsibility, $C_{dev,h}$, for time period *h* by considering their deviations, $q_{dev,h}$, and the prices of the excess, $P_{up,h}$, or lack of, $P_{down,h}$, energy, in cases of up or down deviations, respectively:
  (13) $$\left\{\begin{array}{cc} \;C_{dev,h}=q_{dev,h}P_{up,h}, & \mathrm{for}\;q_{dev,h}>0\\ \;C_{dev,h}=\left|q_{dev,h}\right|P_{down,h}, & \mathrm{for}\;q_{dev,h}<0 \end{array}\right.$$
  Now, we note that a bilateral contract ct corresponds to an energy price, $P_{c_{ct},h}$, and the associated investment, $I_{h}$, of retailers is computed as follows:
  (14) $$I_{h}=\sum_{j=1}^{J}\sum_{ct=1}^{Ct}P_{c_{ct},h}q_{c_{ct},h}+P_{0,h}q_{0,h}+P_{cont,h}q_{cont,j,h}\sum_{s=1}^{S}P_{s,h}q_{s,h}-C_{dev,h}$$
  The profit of retailers per time period is:
  (15) $$R_{h}=\sum_{j=1}^{J}T_{j,h}q_{j,h}-I_{h}$$
  The return of the investment (ROI) is a performance indicator to measure the profitability of investments, and is computed as follows:
  (16) $$ROI=\sum_{h=1}^{H}\frac{R_{h}}{I_{h}}$$
  The following three indicators are used to evaluate the performance of the forecast methods:
  (17) $$\mathrm{MAPE}=\frac{100\%}{H}\sum_{h=1}^{H}\left|\frac{q_{h}-{\hat{q}}_{h}}{q_{h}}\right|$$
  (18) $$\mathrm{NRMSE}=100\%\frac{\sqrt{\frac{1}{H}\sum_{h=1}^{H}{\left({\hat{q}}_{h}-q_{h}\right)}^{2}}}{q_{max}}$$
  (19) $$\mathrm{MAE}=\frac{100\%}{H}\sum_{h=1}^{H}\left|q_{h}-{\hat{q}}_{h}\right|$$
  where $q_{max}$ is the maximum consumption (per time period) associated with the portfolio. MAPE is one of the most used indicators. NRMSE is used to evaluate energy forecasts, but overvalues high errors concerning low errors. MAE indicates the absolute error of forecast methods and also the average demand to be balanced. However, it cannot be used to compare results between different data sets.

## 5. Computational Study

The study considers six competing retailers (software agents). The retailers are assumed to start operating at the Iberian Electricity Market (MIBEL) in 2013. They have a target of 312 real Portuguese consumers for their portfolios, corresponding to around 5% of the total consumption of the country [28]. Table 1 presents the main characteristics of each retailer. Consumers are assumed to be connected to the middle voltage grid, meaning that some of them are part of aggregations of residential and small commercial consumers. They are divided into the following five consumption segments: industrial, large commercial, aggregation of small commercials, aggregation of residential areas, and other aggregations. The time period of the study ranges from 1 January 2012 to 31 December 2013.

Retailers consider the optimization model described in [12] to obtain their optimal portfolios. For 2013, they use the real market prices of 2012, updated with forecasts for 2013, and the real consumption data from 2012, also updated with forecast for 2013, to compute their portfolios. In general, the optimized portfolios only consider a small number of end-use customers.

Retailers offer tariffs to end-use customers with the aim of signing bilateral contracts with them. The tariffs have two terms, either a fixed term, which charges for the power purchase guarantee, and a variable term, which charges for the energy, grid access, global use of the system, and the commercialization part of the tariff. The energy part of the tariff is the only part that can be negotiated with customers. Retailers can offer personalized tariffs to different consumers or the same tariff to consumers inside the same consumption segment. Contract duration is assumed to be twelve months.

Retailers trade energy in the wholesale market only (i.e., we do not consider bilateral contracting to obtain energy sold to customers). We consider a day-ahead market and a hybrid intraday market based on both daily auctions and a continuous procedure, where retailers can adjust their short-term positions 1 h ahead of real-time operation.

To compute the bids to submit to the DAM, forecasts based on the expected weather are considered. Deviations from the bids submitted to the DAM are adjusted in the different sessions of the intraday market. The bids to submit to the IDM are computed by considering the following weights: 0.85 for $w_{x}$, 0.05 for $w_{y}$ and 0.10 for $w_{z}$ (recall last section).

Table 2 and Table 3 show the results of the study. Retailers with portfolios containing a small number of consumers experienced higher errors in their forecasts, indicating that versatility and complementarity between different consumers are important factors to take into account. For instance, retailer $Ret_{1}$ has the smallest number of consumers and the highest forecast errors. We note that a consumption reduction occurred in 2013, meaning that retailers sold energy in the continuous IDM during most hours (see Table 2).

Table 3 shows the expected and real returns of retailers. From 2012 to 2013, we note that the wholesale market prices decreased by around 9.2%, a greater reduction than expected, leading to better outcomes for retailers. Accordingly, the real return was higher than the expected return. By comparing the ROI associated with the participation of retailers in the continuous IDM and the optimal ROI, we conclude that retailers with large portfolios and small forecast errors have smaller differences in these indicators.

The intrinsic variation (IR) computes the difference between the real ROI and the expected $\hat{ROI}$ as follows:

(20) $$IR=\frac{ROI-\hat{ROI}}{\hat{ROI}}\times 100$$

By analysing the IR, we conclude that risk-seeking retailers have a lower increase in expected return, which means that even with higher forecast errors, the portfolios of risk-averse retailers can be considered more stable, leading to better outputs. Additionally, by analysing the MAE, we conclude that the average deviations of retailers are low.

Overall, retailers met more than 90% of the energy needs associated with their portfolios in the DAM. However, they also traded some energy in the intraday market. We note that the extended forecast methods used in this work reduced the errors of retailers and increased their return (when compared with the results presented in [15]). The forecast accuracy of the bids submitted to each intraday session has been significantly improved. Furthermore, all retailers benefited from participating in the continuous intraday market by increasing their return.

## 6. Conclusions

This article described a strategic bidding process for retailers to submit bids to a wholesale market composed by a day-ahead market, an intraday market, and a balancing market. It considered a market design that involves a hybrid model for the intraday market, based on daily auctions and a continuous procedure. The continuous intraday market was used to make adjustments 1 h ahead of real-time operation. The strategic bidding process considered four different hybrid forecast methods: multivariate time series for long-term prediction of electricity prices and consumption, historical meteorological comparison of consumption, short-run trend, and typical consumption behaviour of consumers. The last method was considered for forecasts associated with the continuous intraday market (IDM).

Additionally, the article presented a computational study to illustrate and test both the hybrid market design and the strategic bidding process of retailers, by using real data from the Iberian electricity market for the period 2012–2013. The results confirmed the advantages of considering a continuous intraday market. The continuous IDM significantly improved the forecasts of retailers, providing a good solution to adjust their short-run deviations, without involving energy storage and demand–response programs (although these important aspects can be considered in future work).

The results also indicated important aspects to consider when selecting customers to add to the portfolios of retailers. Risk-seeking retailers showed a behaviour similar to traditional retailers. They considered large portfolios with substantial value at risk and tariffs involving a significant risk premium. Such tariffs seemed to be more competitive that the tariffs proposed by risk-averse retailers. However, risk-averse retailers obtained a better output (ROI) from the market, even with higher demand forecast errors, since their portfolios were more stable (according to both price and consumptions risk).

Future works intend to study how the strategic bidding process can be extended to consider prosumers as part of the portfolio of retailers. Additionally, we aim to study the smooth integration of the continuous intraday market with one or more auctions (or sessions), i.e., we will examine how many auctions should be considered and when they should start.

## Figures and Tables

**Table 1:** Main characteristics of retailer agents (adapted from [15]).

Retailer	Risk Attitude	Pricing Strategy	Tariff Type	Number of Clients	Yearly Energy (GWh)	Expected ROI (%)	VaR (%)
$Ret_{1}$	High aversion	EROP	3-rate	5	2.76	3.75	3.42
$Ret_{2}$	Moderate aversion	ETOMaxR	3-rate	22	475.17	3.95	3.78
$Ret_{3}$	Small aversion	EROP	Single	13	30.46	7.54	3.99
$Ret_{4}$	Small seeking	ERTMC	3-rate	32	290.99	7.93	4.13
$Ret_{5}$	Moderate seeking	ETOMinR	3-rate	13	48.58	7.79	4.19
$Ret_{6}$	High seeking	ETOMinR	3-rate	227	917.03	9.95	4.59

**Table 2:** Experimental results: energy bids and forecast indicators.

Retailer	IDM (Sessions) (%)	MAE (MWh)	MAPE (%)	NRMSE (%)	IDM (Cont.) (%)	Direction (%)	MAE (MWh)	MAPE (%)	NRMSE (%)
$Ret_{1}$	33.08	0.04	15.61	6.75	9.73	−1.57	0.03	8.94	4.23
$Ret_{2}$	27.71	5.77	13.62	5.92	8.55	−1.89	3.68	8.13	4.08
$Ret_{3}$	13.31	0.22	7.02	3.55	7.79	−1.25	0.16	4.86	2.82
$Ret_{4}$	11.79	2.18	7.08	4.45	5.82	−1.26	1.17	3.75	2.37
$Ret_{5}$	12.17	0.28	5.07	3.19	3.46	−0.71	0.19	3.32	2.43
$Ret_{6}$	6.56	2.42	2.43	2.03	1.62	0.18	1.57	1.55	1.35

**Table 3:** Experimental results: market return for retailers.

Retailer	Optimal ROI (%)	ROI (Sessions) (%)	Expected ROI (%)	ROI (cont.) (%)	IR(%)
$Ret_{1}$	6.82	5.24	3.75	5.79	54.40
$Ret_{2}$	7.25	5.98	3.95	6.42	62.53
$Ret_{3}$	11.92	10.97	7.54	11.19	48.41
$Ret_{4}$	12.34	11.36	7.93	11.73	37.20
$Ret_{5}$	9.86	9.16	7.79	9.30	19.38
$Ret_{6}$	11.88	11.51	9.95	11.59	16.48

## Data Availability

Real consumption data for the consumers considered in study can be found at: https://archive.ics.uci.edu/ml/datasets/ElectricityLoadDiagrams20112014#. Iberian market data are available online at: https://www.mercado.ren.pt/EN/Electr/MarketInfo/MarketResults/Pages/default.aspx. Tariffs proposed to consumers and optimal portfolio data can be found at: https://doi.org/10.7910/DVN/WFQ5V0. Observed and forecast meteorological data can be found at http://www.meteomanz.com/index?l=1. Data were accessed on 16 January 2023.

## Abbreviations

CET	Central European Time
CVaR	conditional VaR
DAM	day-ahead market
EROP	Equal Return Optimization
ERTMC	ER Tariff Market-Costs
ETOMaxR	Equal Tariff Optimization at a Maximum Return
ETOMinR	ETO at a Minimum Return
IDM	intraday market
MAE	mean absolute error
MAPE	mean absolute percentage error
MIBEL	Iberian market
MTS	multivariate time series
NRMSE	normalized root mean square error
ROI	return on investment
**Indices**	
$c_{t}$	contract number
*D*	target day
$\hat{D}$	selected past day
*h*	period of the tariff
*i*	period number
*j*	consumer number
*t*	year
*s*	IDM session
**Parameters**	
$c_{j}$	consumer agent
$q_{0,h}$	quantity acquired in the DAM
$q_{c_{c_{t}},h}$	quantity in contracts
$q_{dev,h}$	imbalanced quantity
$q_{j,h}$	consumer consumption
$q_{s,h}$	bid to each IDM session
$q_{max}$	maximum quantity
*C*	retailer’s cost
$q_{t}$	yearly electricity consumption
$I_{h}$	investment
*P*	electricity price
$P_{down,h}$	down deviation price
$P_{up,h}$	up deviation price
$R_{h}$	return
Ret	retailer agent
$T_{j,h}$	tariff
$x_{j,h},y_{j,h},z_{j,h}$	quantity forecasts
*w*	weight

## References

[1] Shahidehpour M., Yamin H., Li Z. (2002). Market Operations in Electric Power Systems.

[2] Lopes F., Coelho H. (2010). Concession Behaviour in Automated Negotiation. E-Commerce and Web Technologies.

[3] Algarvio H. (2023). Risk-Sharing Contracts and risk management of bilateral contracting in electricity markets. Int. J. Electr. Power Energy Syst..

[4] Algarvio H., Lopes F., Couto A., Estanqueiro A. (2019). Participation of Wind Power Producers in Day-ahead and Balancing Markets: An Overview and a Simulation-based Study. Wiley Interdiscip. Rev. Energy Environ..

[5] Lopes F., Coelho H. (2018). Electricity Markets with Increasing Levels of Renewable Generation: Structure, Operation, Agent-Based Simulation and Emerging Designs.

[6] Strbac G., Papadaskalopoulos D., Chrysanthopoulos N., Estanqueiro A., Algarvio H., Lopes F., de Vries L., Morales-España G., Sijm J., Hernandez-Serna R. (2021). Decarbonization of Electricity Systems in Europe: Market Design Challenges. IEEE Power Energy Mag..

[7] Algarvio H., Lopes F., Couto A., Estanqueiro A., Santana J. (2019). Effects of regulating the European internal market on the integration of variable renewable energy. Wiley Interdiscip. Rev. Energy Environ..

[8] EC (2019). Regulation 2019/943. Off. J. Eur. Union.

[9] EC (2019). Directive 2019/944. Off. J. Eur. Union.

[10] Algarvio H., Couto A., Lopes F., Estanqueiro A. (2019). Changing the day-ahead gate closure to wind power integration: A simulation-based study. Energies.

[11] EC (2015). Regulation 2015/1222. Off. J. Eur. Union.

[12] Algarvio H., Lopes F., Sousa J., Lagarto J. (2017). Multi-agent electricity markets: Retailer portfolio optimization using Markowitz theory. Electr. Power Syst. Res..

[13] Algarvio H., Lopes F. (2022). Agent-based Retail Competition and Portfolio Optimization in Liberalized Electricity Markets: A Study Involving Real-World Consumers. Int. J. Electr. Power Energy Syst..

[14] Algarvio H. (2022). Multi-step optimization of the purchasing options of power retailers to feed their portfolios of consumers. Int. J. Electr. Power Energy Syst..

[15] Algarvio H., Lopes F. (2022). Strategic Bidding of Retailers in Wholesale Energy Markets: A Model Using Hybrid Forecast Methods. Highlights in Practical Applications of Agents, Multi-Agent Systems, and Complex Systems Simulation: The PAAMS Collection.

[16] Lopes F., Algarvio H., Santana J. (2017). Agent-based simulation of electricity markets: Risk management and contracts for difference. Agent-Based Modeling of Sustainable Behaviors.

[17] Lopes F. (2018). MATREM: An Agent-based Simulation Tool for Electricity Markets. Electricity Markets with Increasing Levels of Renewable Generation: Structure, Operation, Agent-Based Simulation and Emerging Designs.

[18] Hatami A., Seifi H., Kazem M. (2009). Optimal selling price and energy procurement strategies for a retailer in an electricity market. Electr. Power Syst. Res..

[19] Hatami A., Seifi H., Kazem M. (2011). A Stochastic-Based Decision-Making Framework for an Electricity Retailer: Time-of-Use Pricing and Electricity Portfolio Optimization. IEEE Trans. Power Syst..

[20] Kettunen J., Salo A., Bunn D. (2010). Optimization of electricity retailer’s contract portfolio subject to risk preferences. IEEE Trans. Power Syst..

[21] Nojavan S., Nourollahi R., Pashaei-Didani H., Zare K. (2019). Uncertainty-based electricity procurement by retailer using robust optimization approach in the presence of demand response exchange. Int. J. Electr. Power Energy Syst..

[22] Ayón X., Gruber J., Hayes B., Usaola J., Prodanovic M. (2017). An optimal day-ahead load scheduling approach based on the flexibility of aggregate demands. Appl. Energy.

[23] Wei N., Li C., Peng X., Zeng F., Lu X. (2019). Conventional models and artificial intelligence-based models for energy consumption forecasting: A review. J. Pet. Sci. Eng..

[24] Koponen P., Ikäheimo J., Koskela J., Brester C., Niska H. (2020). Assessing and comparing short term load forecasting performance. Energies.

[25] Shah I., Iftikhar H., Ali S. (2022). Modeling and forecasting electricity demand and prices: A comparison of alternative approaches. J. Math..

[26] Aven T., Renn O. (2010). Risk Management and Governance: Concepts, Guidelines and Applications.

[27] Hopkin P. (2012). Fundamentals of Risk Management: Understanding, Evaluating and Implementing Effective Risk Management.

[28] Rodrigues F., Trindade A. (2018). Load forecasting through functional clustering and ensemble learning. Knowl. Inf. Syst..

